# Hypertension among Mississippi Workers by Sociodemographic Characteristics and Occupation, Behavioral Risk Factor Surveillance System

**DOI:** 10.1155/2020/2401747

**Published:** 2020-07-16

**Authors:** Vincent L. Mendy, Rodolfo Vargas, Oluwabunmi Ogungbe, Lei Zhang

**Affiliations:** ^1^Department of Epidemiology and Biostatistics, School of Public Health, Jackson State University, 350 W. Woodrow Wilson Dr., Suite 2300, Jackson, MS 39123, USA; ^2^Office of Health Data and Research, Mississippi State Department of Health, 570 E. Woodrow Wilson, P.O. Box 1700, Jackson, MS 39215, USA; ^3^Johns Hopkins School of Nursing, 525 N. Wolfe Street, Baltimore, MD 21205, USA

## Abstract

In 2017, Mississippi had the third highest age-adjusted prevalence of hypertension in the United States. We estimated the prevalence of hypertension by sociodemographic characteristics and occupation and examined the association between hypertension with occupation and sociodemographic characteristics among Mississippi workers. We calculated adjusted prevalence and adjusted prevalence ratios (APRs) by sociodemographic characteristics and occupation among Mississippi adult workers. We analyzed combined 2013, 2015, and 2017 data from the Mississippi Behavioral Risk Factor Surveillance System for 6,965 workers in ten Standard Occupational Classification System major groups. Of the estimated 1.1 million Mississippi workers during the three survey years, 31.4% (95% confidence interval (CI), 30.0–32.8) had hypertension. The likelihood of having hypertension was significantly higher among workers aged 30–44 years, 45–64 years, blacks, and those classified as overweight and obese workers compared to their counterparts. The likelihood of having hypertension among workers in the fields of installation, repair and maintenance, and production were 26% higher (APR, 1.26; 95% CI, 1.03–1.55) and 33% higher (APR, 1.33; 95% CI, 1.11–1.58), respectively, than workers in all other occupational groups. Among Mississippi workers, hypertension prevalence varied by sociodemographic characteristics and occupational groups. Age, race, obesity status, installation, repair, maintenance, and production occupation groups are associated with an increased likelihood of hypertension. Novel and/or community-based or linked programs are needed that could target workers at risk of hypertension that are outside of a single-site workplace.

## 1. Introduction

In 2017, Mississippi had the third highest age-adjusted prevalence (38.2%) of hypertension in the United States (US) [1]. Hypertension is a major risk factor for cardiovascular disease (CVD) and stroke [2]. In 2018, heart disease and stroke were the first and sixth leading causes of death in Mississippi, respectively [3]. Untreated or uncontrolled hypertension is the single largest contributor to CVD [4], which is the leading cause of death in Mississippi [5]. Work environment and work stress are associated with both ischemic heart disease [6] and coronary heart disease [7]. A prior epidemiological study has documented association between hypertension and occupation in US workers [8]. Data on hypertension and occupation among Mississippi workers are limited.

Understanding hypertension among the Mississippi workforce will facilitate the development of effective workplace promotion and intervention programs as well as work health related programs. We estimated the prevalence of hypertension by sociodemographic characteristics and occupation. Here, we report the estimation of the association between hypertension and occupation and sociodemographic characteristics among Mississippi workforce.

## 2. Methods

We analyzed combined data from the 2013, 2015, and 2017 Mississippi Behavioral Risk Factor Surveillance System (BRFSS), which included an industry and occupation module. The BRFSS is a state-based telephone survey of the U S noninstitutionalized civilian population aged 18 years or older. The survey was conducted in all 50 states, the District of Columbia, and the U S territories. Data from the BRFSS provide reliable and valid assessments of health risk factors [9] Detailed information about BRFSS is available at http://www.cdc.gov/brfss/. The Jackson State University Institutional Review Board deemed this study exempt from review.

Mississippi began collecting data via the optional industry and occupation module in 2012, but data on hypertension were only available in odd years. Survey respondents who were employed for wages or self-employed were asked, “What kind of business or industry do you work in?” and “What kind of work do you do?” Answers were open-ended and were coded using the 574 Census Bureau (2002) occupational numeric codes. For analysis, these codes were grouped into ten Standard Occupational Classification System major groups [10]. The current analyses were restricted to respondents who self-identified as black or white; which accounted for 96.6% of the Mississippi population in 2010 [3]. We excluded workers who were on active military duty (*n* = 26) as well as those missing information about employment (*n* = 852). The final analytic sample included 6,965 respondents.

High blood pressure was defined as a “yes” response to the question, “Have you ever been told by a doctor, nurse, or other health professional that you have high blood pressure?” Normal weight, overweight, and obesity were defined as having a body mass index (BMI) of 18.5–<25.0, 25.0–<30.0, and ≥30.0 kg/m^2^, respectively (calculated from self-reported height and weight).

### 2.1. Statistical Analyses

Adjusted prevalence and 95% confidence intervals (CIs) were calculated for hypertension prevalence overall and by sociodemographic characteristics and occupation, adjusting for age, sex, race, income, education, BMI, and occupation. In addition, logistic regression was used to calculate adjusted prevalence ratios (APRs) for hypertension in relation to sociodemographic characteristics and occupation. We used SAS 9.4 (SAS Institute, Inc.) and SUDAAN 11 to adjust for the disproportionate stratified sampling design of BRFSS; since 2011 a new statistical method called raking has been used to weight the BRFSS data. It included additional population characteristics such as educational level, marital status, and home ownership status of respondents [11]. We used a significance threshold of *p* < 0.05.

## 3. Results

There were an estimated 1,073,235 adult workers in Mississippi during the three survey years (2013, 2015, and 2017). Of these workers, 37.6% (95% CI, 36.0–39.3) were aged 30–44 years, 37.5% (95% CI, 36.0–39.0) were aged 45–64 years, 54.2% (95% CI, 52.6–55.8) were male, 37.5% (95% CI, 35.9–39.1) were black, 61.6% (95% CI, 60.1–63.4) had greater than a high school education, 40.2% (95% CI, 38.5–41.9) had an annual household income of less than $35,000, 36.2% (95% CI, 34.6–37.8) were classified as obese, and 22.8% (95% CI, 21.4–24.3) were current smokers (Table 1).

The overall adjusted prevalence of hypertension among Mississippi workers was 31.4% (95% CI, 30.0–32.8). Men (32.3%; 95% CI, 30.2–34.4) had a higher adjusted prevalence than women (30.3%; 95% CI, 28.4–32.2). Adjusted prevalence of hypertension was higher in black workers (33.3%; 95% CI, 30.8–35.8) than white workers (30.2%; 95% CI, 28.5–32.0). Workers aged 45–64 years (47.5%; 95% CI, 45.4–49.7), those with less than a high school education (39.3%; 95% CI, 33.7–45.0), those with an annual household income of $35,000–$49,999 (34.1%; 95% CI, 30.1–38.1), and those with a BMI of ≥30.0 (44.4%; 95% CI, 41.8–47.1) had the highest adjusted prevalence of hypertension in their respective groups, and hypertension prevalence among current working smokers was 32.0% (95% CI, 28.7–35.4) (Table 1). Among the ten occupation categories, the following four occupation groups had the highest adjusted prevalence of hypertension: transportation and material moving (40.0%; 95% CI, 33.5–46.4); production (39.9%; 95% CI, 33.3–46.5); installation, repair, and maintenance (e.g., electric repairs, air-conditioning and heating system repairs, plumbing, painting, flooring, and mopping) (38.0%; 95% CI, 30.2–45.8); and management, business, and financial operations (37.4%; 95% CI, 32.8–42.0) (Table 2).

Relative to workers aged 18–29 years, the likelihood of hypertension among workers aged 30–44 years was 1.98 times higher (APR, 1.98; 95% CI, 1.46–2.68) and the likelihood among workers aged 45–64 years was 3.96 times higher (APR, 3.96; 95% CI, 2.97–5.28). The likelihood of hypertension was 19% higher (APR, 1.19; 95% CI, 1.06–1.33) among black workers than among white workers. Compared to workers with a normal weight, overweight workers had a 69% higher (APR, 1.69; 95% CI, 1.41–2.02) likelihood of hypertension while the likelihood of hypertension among obese workers was 2.56 times higher (APR, 2.56; 95% CI, 2.17–3.03). Compared to workers in all other occupations, installation, repair, and maintenance workers (automatic service technicians and mechanics; general maintenance and repair workers; heat, air-conditioning, and refrigeration mechanics and installers) had a 26% higher (APR, 1.26; 95% CI, 1.03–1.55) likelihood of hypertension, while workers in production (e.g., poultry, assemblers and fabricators, bakers, butchers and meat cutters, rigging, metal and plastic workers, and plant and system operators) had a 33% higher (APR, 1.33; 95% CI, 1.11–1.58) likelihood of hypertension (Table 3).

## 4. Discussion

An estimated 3 out of every 10 Mississippi workers had hypertension during the three survey years. Among the Mississippi work force, the prevalence of hypertension differed by occupation and sociodemographic characteristics. Age, race, and obesity status were associated with a higher prevalence of hypertension, as were specific occupational categories, such as installation, repair, maintenance, and production occupation groups.

The increased likelihood of having hypertension by age, race, and obesity status is consistent with previous research [12]. In addition, in the Jackson Heart Study cohort, mean systolic blood pressure and diastolic blood pressure levels increased with age [13], and obesity is associated increased incidence of hypertension [14]. The higher likelihood of hypertension among these subgroups may be partially due to an increasing proportion of Mississippi adults in the two highest-risk BMI categories (obesity, BMI ≥30; extreme obesity, BMI ≥40) [15] and a disproportionately higher prevalence of hypertension risk factors (e.g., physical inactivity and diabetes) among these groups [1]. In 2017, among Mississippi adults, diabetes prevalence was 13.3% for whites and 16.0% for blacks and 1.3% among those aged 18–24 years and 25.0% among those aged 55–64 years [16]. In addition, 31.6% of white adults reported not participating in any physical activity outside of work in the past 30 days compared to more than one-third (36.0%) among black adults.

The higher likelihood of hypertension among installation, repair, and maintenance workers could be due to occupational strain or stress [17] or long work hours [18]. In the Multiethnic Study of Atherosclerosis (MESA) study, among participants working more than 20 hours per week, Landsbergis et al., (2015) found that lower job decision latitude (“job control”) was associated with hypertension in several occupations [19]. In a prospective study of Canadian white collar workers, exposure to cumulative job strain had a modest but significant effect on systolic blood pressure among men. Those with low levels of social support at work had higher risk for increases in blood pressure [20]. A recent study also showed that long work hours increases blood pressure [21]. This could help explain our finding of increased likelihood of hypertension among Mississippi production workers. Production occupation exposure, including shiftwork and noise exposure, is shown to have additive effect of the occurrence of hypertension [22]. Employer-based workplace health promotion programs can lower the prevalence of chronic conditions such as hypertension and improve the health and well-being of workers [23].

BRFSS uniquely provides state-level estimates of occupation-specific hypertension prevalence among Mississippi adults. Because the BRFSS consists of self-reported information, the data are subject to recall bias and social desirability bias [24]. In addition, because the data are cross-sectional, we cannot make causal inferences based on the results.

## 5. Conclusion

Older adults, blacks, those classified as overweight and obese, and those in the fields of installation, repair and maintenance, and production could benefit from workplace prevention and health promotion programs focused on hypertension. There is a need for novel and/or community-based or linked programs that could target individuals at risk that are outside of a single-site workplace, potentially using mHealth and eHealth interventions and/or supporting the promotion of a medical home and/or regular visits with a healthcare provider to identify those at risk.

## Figures and Tables

**Table 1:** Sociodemographic characteristics of Mississippi adult workers (*N* = 6,965), 2013, 2015, and 2017 BRFSS.

Characteristic	*n* ^a^	*N* ^b^	%^C^ (95% CI)
*Age group*, *years*			
18–29	832	253846	24.9 (23.2–26.5)
30–44	1832	384010	37.6 (36.0–39.3)
45–64	3522	382236	37.5 (36.0–39.0)
*Sex*			
Male	3094	581918	54.2 (52.6–55.8)
Female	3871	491317	45.8 (44.2–47.4)
*Race*			
Black	2415	402821	37.5 (35.9–39.1)
White	4551	670421	62.5 (60.9–64.1)
*Education level*			
<high school graduate	442	112409	10.5 (9.3–11.7)
High school graduate or equivalent	1767	297591	27.8 (26.3–29.2)
>high school graduate	4749	662322	61.8 (60.1–63.4)
*Annual household income*, $			
<35,000	2206	386742	40.2 (38.5–41.9)
35,000–49,999	972	148759	15.5 (14.2–16.7)
≥50,000	3077	426532	44.3 (42.7–46.0)
*Body mass index (kg/m* ^*2*^)			
18.0–<25.0	1644	267779	26.8 (25.3–28.3)
25.0–<30.0	2434	369735	37.0 (35.4–38.6)
≥30	2405	361703	36.2 (34.6–37.8)
*Current smoker*			
Yes	1239	234494	22.8 (21.4–24.3)
No	5478	792130	77.2 (75.7–78.6)

CI, conference interval; BRFSS, Behavioral Risk Factor Surveillance System. ^a^Unweighted number. ^b^Weighted number. ^c^Weighted percentage.

**Table 2:** Adjusted prevalence of blood pressure among Mississippi adult workers by sociodemographic characteristics and occupation, 2013, 2015, and 2017 BRFSS.

Occupational groups	Adjusted prevalence (%)^a^	95% CI
Overall	31.4	30.0–32.8
*Age group*, *years*		
18–29	11.0	8.5–13.6
30–44	25.1	22.6–27.6
45–64	47.5	45.4–49.7
*Sex*		
Male	32.3	30.2–34.4
Female	30.3	28.4–32.2
*Race*		
Black	33.3	30.8–35.8
White	30.2	28.5–32.0
*Education level*		
<high school graduate	39.3	33.7–45.0
High school graduate or equivalent	31.6	28.9–34.2
>high school graduate	30.0	28.2–31.7
*Annual household income*, $		
<35,000	31.2	28.7–33.7
35,000–49,999	34.1	30.1–38.1
≥50,000	31.8	29.6–33.9
*Body mass index (kg/m* ^*2*^)		
18.0–<25.0	16.2	14.0–18.4
25.0–<30.0	30.1	27.6–32.5
≥30	44.4	41.8–47.1
*Current smoker*		
Yes	32.0	28.7–35.4
No	68.0	64.6–71.3
*Occupation group*		
Management, business, and financial operations	37.4	32.8–42.0
Professional and related occupations	29.3	26.6–32.1
Service occupations	29.6	26.0–33.2
Sales and related	29.5	24.6–34.3
Office and administrative support	28.7	24.5–33.0
Construction and extraction	24.1	18.2–30.0
Installation, repair, and maintenance	38.0	30.2–45.8
Production	39.9	33.3–46.5
Transportation and material moving	40.0	33.5–46.4

CI, confidence interval; BRFSS, Behavioral Risk Factor Surveillance System. ^a^Adjusted for age, sex, education, annual household income, body mass index, and smoking.

**Table 3:** Adjusted prevalence ratios of hypertension in relation to sociodemographic characteristics and occupation among Mississippi adult workers, 2013, 2015, and 2017 BRFSS.

Occupational groups	Adjusted prevalence ratio^a^	95% CI
*Age group*, *years*		
18–29	Reference	
30–44	1.98	1.46–2.68
45–64	3.96	2.97–5.28
*Sex*		
Male	1.06	0.94–1.19
Female	Reference	
*Race*		
Black	1.19	1.06–1.33
White	Reference	
*Education level*		
<high school graduate	1.14	0.93–1.40
High school graduate or equivalent	0.95	0.84–1.08
>high school graduate	Reference	
*Annual household income*		
<35,000	1.08	0.94–1.23
35,000–49,999	1.14	0.98–1.32
≥50,000	Reference	
*Body mass index (kg/m* ^*2*^)		
18.0–<25.0	Reference	
25.0–<30.0	1.69	1.41–2.02
≥30	2.56	2.17–3.03
*Current smoker*		
Yes	1.10	0.98–1.25
No	Reference	
*Occupation group*		
Management, business, and financial operations	1.11	0.95–1.29
Professional and related occupations	0.96	0.85–1.09
Service occupations	0.91	0.79–1.06
Sales and related	0.95	0.79–1.14
Office and administrative support	0.93	0.78–1.10
Construction and extraction	0.77	0.57–1.03
Installation, repair, and maintenance	1.26	1.03–1.55
Production	1.33	1.11–1.58
Transportation and material moving	0.96	0.78–1.19
All other occupational groups	Reference	

CI, confidence interval; BRFSS, Behavioral Risk Factor Surveillance System. ^a^Adjusted for age, sex, education, annual household income, body mass index, and smoking.

## Data Availability

Data are available from the Mississippi State Department of Health for researchers who meet the criteria for data approval for research.
